# Novel HER2-targeted therapy to overcome trastuzumab resistance in *HER2*-amplified gastric cancer

**DOI:** 10.1038/s41598-023-49646-5

**Published:** 2023-12-19

**Authors:** Juin Park, Sun Kyoung Kang, Woo Sun Kwon, Inhye Jeong, Tae Soo Kim, Seo Young Yu, Sang Woo Cho, Hyun Cheol Chung, Sun Young Rha

**Affiliations:** 1 Song-Dang Institute for Cancer Research, Yonsei University College of Medicine, Seoul, 03722 Republic of Korea; 2 Department of Medicine, Yonsei University College of Medicine, Seoul, 03722 Republic of Korea; 3 Brain Korea 21 PLUS Project for Medical Science, Yonsei University College of Medicine, Seoul, 03722 Republic of Korea; 4 Yonsei Cancer Center, Yonsei University Health System, Seoul, 03722 Republic of Korea; 5 Division of Medical Oncology, Department of Internal Medicine, Yonsei University College of Medicine, Seoul, 03722 Republic of Korea

**Keywords:** Cancer, Cell biology, Molecular biology

## Abstract

Trastuzumab is used to treat *HER2*-amplified metastatic gastric cancer; however, most patients become trastuzumab-resistant within a year. Knowledge of the mechanisms underlying trastuzumab resistance is required to overcome this limitation. Here, we aimed to elucidate this resistance mechanism using four trastuzumab-resistant (TR) cell lines and investigate the efficacy of HER2-targeted therapies to overcome treatment resistance. Each TR cell line had different phenotypic characteristics. Interestingly, HER2 expression remained as high as the parental cell lines in TR cell lines, suggesting that HER2-targeted agents were still useful. As expected, three tyrosine kinase inhibitors (lapatinib, neratinib, and tucatinib) and one antibody–drug conjugate (trastuzumab deruxtecan: T-DXd) exhibited good antitumor effects against TR cell lines. We further investigated the potential biological mechanism of T-DXd. When treated with trastuzumab or T-DXd, HER2 or its downstream signals were disrupted in parental cell lines, but not in TR cell lines. Moreover, T-DXd induced the expression of pH2A.X and cPARP and caused cell cycle arrest in the S or G2-M phase in TR cell lines. T-DXd showed promising antitumor activity in both parental and TR cell lines, suggesting that it is a potential candidate for overcoming trastuzumab resistance.

## Introduction

Gastric cancer (GC) is the fifth most frequently diagnosed cancer and third among the leading causes of cancer-related death worldwide^1^. Despite the progress in its management, the prognosis of advanced or metastatic GC remains poor, with a 5-year survival rate lower than 10%^2^. Thus, the search for effective and novel therapies remains a challenge.

HER2/Neu, a member of the ErbB family of receptor tyrosine kinases (RTKs), has received considerable attention as a promising therapeutic target for various cancers, including GC^3^. Typically, HER2 overexpression by 10–25% in GC is associated with a poor prognosis^4^. Trastuzumab is a targeted drug that binds to the extracellular domain IV of HER2 and inhibits its dimerization, thereby preventing HER2-mediated signals and inducing antibody-dependent cellular cytotoxicity^5^. In January 2010, the U.S. Food and Drug Administration (FDA) approved trastuzumab for the treatment of HER2-overexpressing gastric and gastroesophageal junction cancers; currently, it is considered a representative targeted therapy^6^. Trastuzumab in combination with chemotherapy increased the survival rate of patients compared to that by chemotherapy alone^7^. However, most patients who initially responded to trastuzumab developed acquired resistance within a year. Therefore, knowledge about alternative drugs and their mechanisms of action is crucial to overcome resistance to trastuzumab-based therapies^8,9^.

Several HER2-targeted drugs have been developed over the last few years, including tyrosine kinase inhibitors (TKIs) and antibody–drug conjugates (ADCs)^10–13^. To date, three HER2-targeted TKIs have been approved by the FDA for the treatment of HER2-positive breast cancer: a dual EGFR/HER2 inhibitor lapatinib, pan-HER inhibitor neratinib, and selective HER2 inhibitor tucatinib^14,15^, which are not used in GC. ADCs are a new class of potent therapeutic drugs that combine the targeting ability of a monoclonal antibody with a cytotoxic agent through a linker^16^. Trastuzumab deruxtecan (T-DXd) is a novel HER2-targeted ADC comprising an anti-HER2 antibody, a cleavable peptide-based linker, and a cytotoxic topoisomerase I inhibitor, an exatecan derivative^17^. T-DXd was approved by the FDA in January 2021 for the treatment of previously treated patients with HER2-positive metastatic gastric or gastroesophageal junction adenocarcinoma^18^. The biological mechanism of T-DXd in HER2 overexpression and moderate expression cell models has been investigated in several studies^17,19,20^; however, its efficacy and biological mechanism in trastuzumab-resistant (TR) cell lines were not studied.

Therefore, this study aimed to elucidate the underlying mechanisms of acquired trastuzumab resistance in vitro and investigate the efficacy of HER2-targeted therapies in overcoming trastuzumab resistance in *HER2*-amplified GC cell lines. We illustrated a novel perspective of the differential biological activities of T-DXd in parental and TR cell lines.

## Materials and methods

### Cell lines

Six *HER2*-amplified GC cell lines were used in this study. NCI-N87 (RRID: CVCL_1603), SNU-216 (RRID: CVCL_3946), and MKN-7 (RRID: CVCL_1417) cell lines were purchased from the American Type Culture Collection (Manassas, VA, USA), Korean Cell Line Bank (Seoul, Republic of Korea), and Japanese Cancer Research Resources Cell Bank (Osaka, Japan), respectively. YCC-19, YCC-33, and YCC-38 cell lines were established by the Song-Dang Institute for Cancer Research (SICR) Cell Line Bank (Seoul, Republic of Korea). As control cells for HER2 expression, three breast cancer cell lines (SK-BR-3, ZR-75–1, and MCF7) were used, which were purchased from the American Type Culture Collection. The cells were cultured in Eagle’s Minimum Essential Medium or RPMI-1640 medium containing 10% fetal bovine serum (Lonza Inc., Basel, Switzerland), 100 units/mL of penicillin, and 100 mg/mL of streptomycin (Lonza Inc.). The cultured cells were incubated at 37 °C in a humidified atmosphere with 5% CO_2_.

### Evaluation of HER2 status

HER2 status was obtained from the SICR database, including targeted sequencing for copy number variation, RNA sequencing for RNA expression analysis^21–23^, protein expression by immunohistochemistry (IHC) analysis, and gene amplification by silver in situ hybridization.

### Reagents

Trastuzumab was provided by Celltrion Inc. (Incheon, Republic of Korea). Tucatinib, lapatinib, neratinib, and SN-38 were purchased from Selleckchem, Inc. (Houston, TX, USA). T-DXd provided by Daiichi Sankyo Co., Ltd. (Tokyo, Japan) was used for in vitro experiments.

### Establishment of acquired TR GC cell lines

Acquired TR GC cell lines were established by culturing six GC cell lines with amplified *HER2* in the presence of serially increasing concentrations of trastuzumab^24^. This incubation was continued with concentrations of trastuzumab starting at 100 μg/mL and reaching 3200 μg/mL; the drug concentration was doubled after every five passages. Acquired TR cell lines established after 30 passages were maintained with 200 μg/mL of trastuzumab, the minimum dose required to maintain resistance. Acquired TR cell lines were established when there was at least a 20% inhibition difference at 200 μg/mL of trastuzumab compared to that in the respective parental cell lines.

### Transwell migration and invasion assays

Transwell migration assay was performed following methods described previously^25^. Briefly, 0.5 × 10^5^–1 × 10^6^ cells were resuspended in serum-free medium and seeded into the upper chamber of transwell culture plates. The lower chambers of the plates were filled with complete medium containing 10% serum. After 24 h of incubation, migrated cells were fixed using 4% formaldehyde and stained with 0.5% crystal violet solution. The cells were observed using an optical microscope, and the microscopy images were analyzed using ImageJ software (https://imagej.nih.gov/ij/). For the invasion assay, the upper surface of the membrane was covered with Matrigel (Corning Inc., MA, USA) for 4 h, and subsequently, the experiment was processed as described for the migration assay.

### Cell proliferation and viability assays

For the cell proliferation assay, 5 × 10^3^ cells were seeded into 24-well plates and cultured for 7 days. Cell growth was measured at 24-h intervals using an assay based on the colorimetric conversion of 3-(4,5-dimethlthiazol-2-yl)-2,5-diphenyltetrazolium bromide (Sigma-Aldrich Inc., St. Louis, MO, USA). The absorbance was measured at 570 nm using an absorbance microplate reader (Tecan Inc., Mannedorf, Switzerland). For the cell viability assay, 2 × 10^3^–5 × 10^3^ cells were seeded into 96-well plates and treated with various concentrations of the drugs for different durations. Cell viability was assessed using the Cell Counting Kit-8 assay (Dojindo Inc., Kumamoto, Japan) as described previously^21^. Absorbance was measured at 450 nm using an absorbance microplate reader (Tecan Inc.). Cell viability was calculated relative to that in the control condition. The IC50 was determined using Calcusyn.

### Flow cytometry analysis

Cells (2 × 10^5^–5 × 10^5^) were seeded into 60-mm dishes to quantify HER2 expression on the cell surface and to assess the cell cycle distribution. After 24 h, the cells were treated with T-DXd or trastuzumab for 72 h and were harvested. For quantification of HER2 expression, 3 × 10^5^ cells were stained with APC-labeled anti-human CD340 (erbB2/HER-2) antibody (3:100, #324,408; BioLegend, Inc., San Diego, CA, USA) and APC-labeled anti-mouse IgG1, κ Isotype Ctrl antibody (3:100, #400,122; BioLegend, Inc.) at 4 °C for 15 min in the dark. After washing twice with FACS buffer, the cells were resuspended in 200 μL of FACS buffer. Three breast cancer cell lines—SK-BR-3 (3 +), ZR-75–1 (2 +), and MCF7 (1 +)—were used as controls of HER2 expression, representing different HER2 levels (indicated in parentheses). To assess the cell cycle distribution, the cells were fixed for at least 24 h in 75% cold ethanol as described previously and then stained with propidium iodide/RNase Staining Buffer (BD Biosciences Inc., Franklin Lakes, NJ, USA) for 15 min in the dark^26^. Data were analyzed using BD LSRII (BD Biosciences Inc.) and FlowJo software (Tristar Inc., CA, USA).

### Immunoblotting analysis

Total protein was extracted from the cell lines following the standard protocol, and 20–40 μg of protein was used for immunoblotting^27^. The blots were cut prior to hybridization with antibodies during blotting. The primary antibodies purchased from Cell Signaling Technology, Inc. (Danvers, MA, USA) were as follows: pHER2-Tyr1221/1222 (#2249), pEGFR-Tyr1173 (#4407), EGFR (#2232), pHER3-Tyr1289 (#4791), pMET-Tyr1234/1235 (#3077), pIGF1R-Tyr1135/1136 (#3024), IGF1R (#9750), ERK (#4695), pAKT-Ser473 (#4060), pAKT-Thr308 (#4056), AKT (#9272), pH2A.X-Ser139 (#9718), cleaved PARP (#9541), and PCNA (#2586). Other primary antibodies [HER3 (sc-285), MET (sc-514148), pERK-Tyr204 (sc-7383), and PTEN (sc-6818)] were purchased from Santa Cruz Biotechnology, Inc. Anti-HER2 (ab16901) and anti-α-tubulin (T6199) antibodies were purchased from Abcam Inc. and Sigma-Aldrich, Inc., respectively. After incubation with peroxidase-conjugated secondary antibodies for 1 h at 20 °C, the protein blots were developed using an enhanced chemiluminescent reagent (Amersham Inc., Amersham, UK). Total protein visualized on the ChemiDoc™ XRS + System (Bio-Rad) and analysed on Image Lab software (Image Lab 6.0, Bio-Rad).

### Statistical analysis

The results are presented graphically as the mean ± standard deviation of the mean. GraphPad Prism software was used for the statistical analyses. Two-way ANOVA was used for statistical analysis followed by Bonferroni’s post-hoc test. All data were analyzed using the Student’s t-test, with *p*-values < 0.05 (*p < 0.05, **p < 0.01, ***p < 0.001) indicating statistical significance, whereas NS indicated not statistically significant.

## Results

### Establishment of TR GC cell lines

We previously screened the genomic patterns of several RTKs (EGFR, HER2, MET, FGFR2, and IGF1R) in a panel of 49 GC cell lines, and *HER2* amplification was observed in six cell lines^21^. To clearly identify the HER2 status in these cell lines, we confirmed *HER2* amplification at the DNA level and overexpression at the RNA and protein levels using the SICR database (Table 1 and Supplementary Fig. S1).

**Table 1:** Integrative profiling of HER2 status of GC cell lines by targeted DNA sequencing, RNA sequencing, silver in situ hybridization, immunohistochemistry, and flow cytometry.

Cell line	CNV	RNA expression (FPKM)	HER2/CEP17 ratio	IHC Score	MFI
YCC-19	24.4	6282.9	25	3 +	7040.30
NCI-N87	18.1	1934.3	10	3 +	5095.89
YCC-38	12.5	2306.7	10	3 +	3340.49
YCC-33	11.2	847.6	4.5	3 +	6803.54
MKN-7	6.7	1546.8	3.7	2 +	1064.70
SNU-216	2.7	424.6	4.4	2 +	792.30
MKN-45	0.8	73.1	NA	0	105.83

Abbreviations: CNV, copy number variation; FPKM, fragments per kilobase of transcripts per million mapped reads; HER2, human epidermal growth factor receptor 2; CEP-17, centromere enumerator probe 17; IHC, immunohistochemistry; MFI, mean fluorescence intensity.

**Figure 1:**
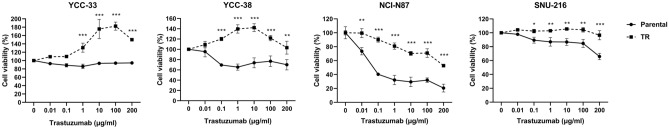
Trastuzumab sensitivity of parental and trastuzumab-resistant (TR) cell lines. Parental and TR cell lines were treated with serial concentrations of trastuzumab for 6 days. Cell viability was measured using the Cell Counting Kit-8 assay. Error bars, SD. ***p < 0.001, **p < 0.01, *p < 0.05.

To understand the mechanisms underlying trastuzumab resistance, we established TR cell lines using six *HER2*-amplified GC cell lines. Four TR cell lines were established: YCC-33TR, YCC-38TR, NCI-N87TR, and SNU-216TR. The parental cell lines were sensitive to trastuzumab, whereas four TR cell lines developed a significant level of resistance to trastuzumab (*p* < 0.05; Fig. 1). In detail, the difference in trastuzumab inhibition rate at 200 μg/mL between the parental and TR cell lines was the following: 55.7% (YCC-33TR), 33.1% (YCC-38TR), 32.1% (NCI-N87TR), and 30.7% (SNU-216TR). In contrast, induction of trastuzumab resistance in YCC-19 and MKN-7 cells failed, since the establishment criteria were not met (data not shown).

### Comparison of the phenotypic and molecular characteristics of parental and TR cell lines

**Figure 2:**
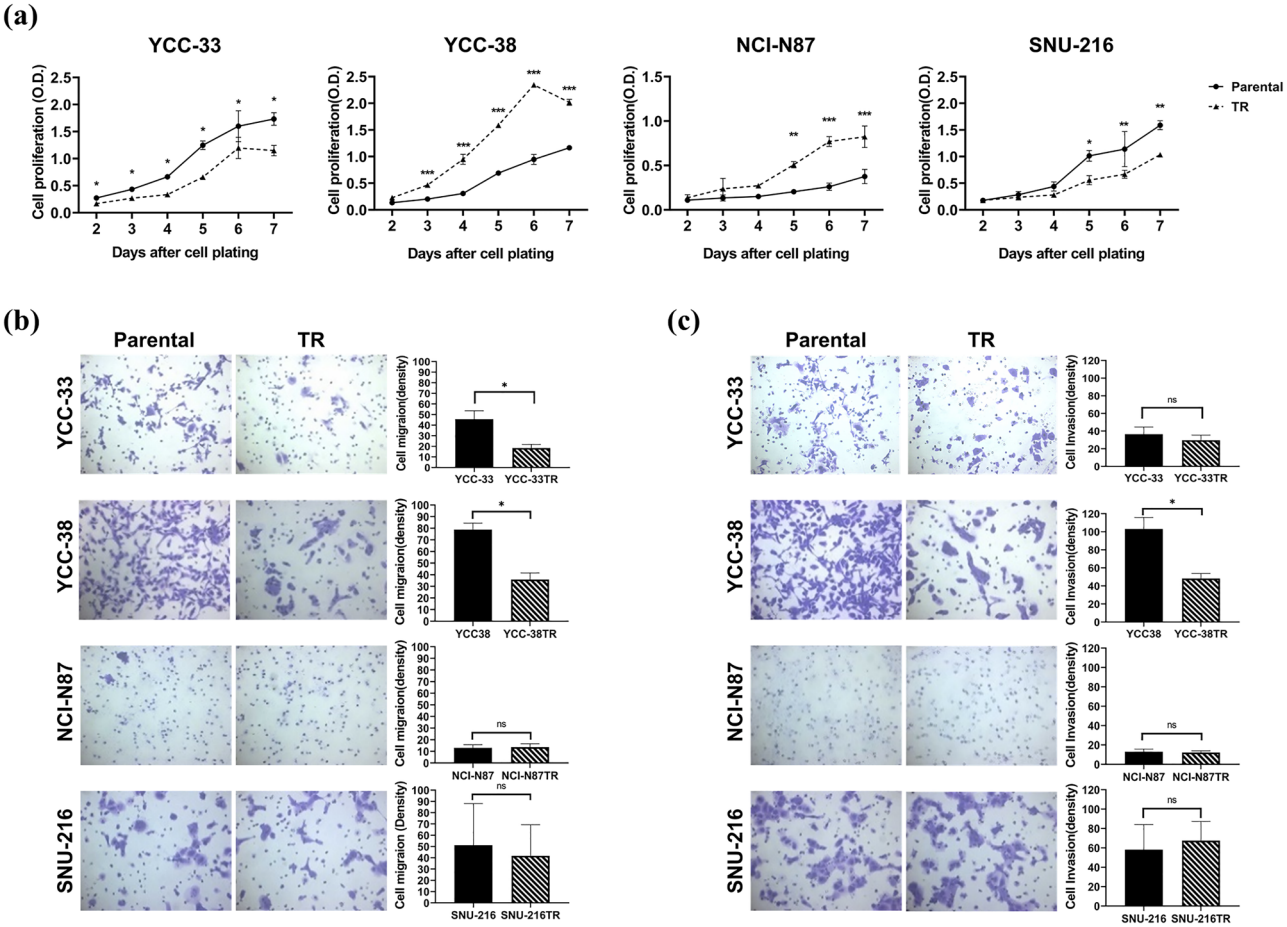
Comparison of phenotypic changes between parental and trastuzumab-resistant (TR) cell lines. (**a**) Proliferation activities were measured daily via the MTT assay. (**b**) Migration capacities were measured by transwell assay and quantified using ImageJ software. (**c**) Invasion capacities were measured by transwell assay and quantified using ImageJ software. ***p < 0.001, **p < 0.01, *p < 0.05, and ns: no significant difference.

For the phenotypic characterization of TR cell lines, their proliferation, migration, and invasion capacities were investigated. We evaluated and compared the proliferative capacities of the parental and TR cell lines. Compared to the respective parental cell lines, YCC-33TR and SNU-216TR grew significantly slower, whereas YCC-38TR and NCI-N87TR grew more rapidly (*p* < 0.05; Fig. 2a). The changes in migration and invasion ability were evaluated using a transwell assay. Compared with the respective parental cell lines, YCC-33TR had significantly reduced migration ability by 0.4-fold, and YCC-38TR had significantly reduced migration and invasion ability by 0.5-fold. NCI-N87TR and SNU-216TR did not differ significantly from the parental cells (Fig. 2b,c). These results demonstrated that each TR cell line developed different phenotypic features.

**Figure 3:**
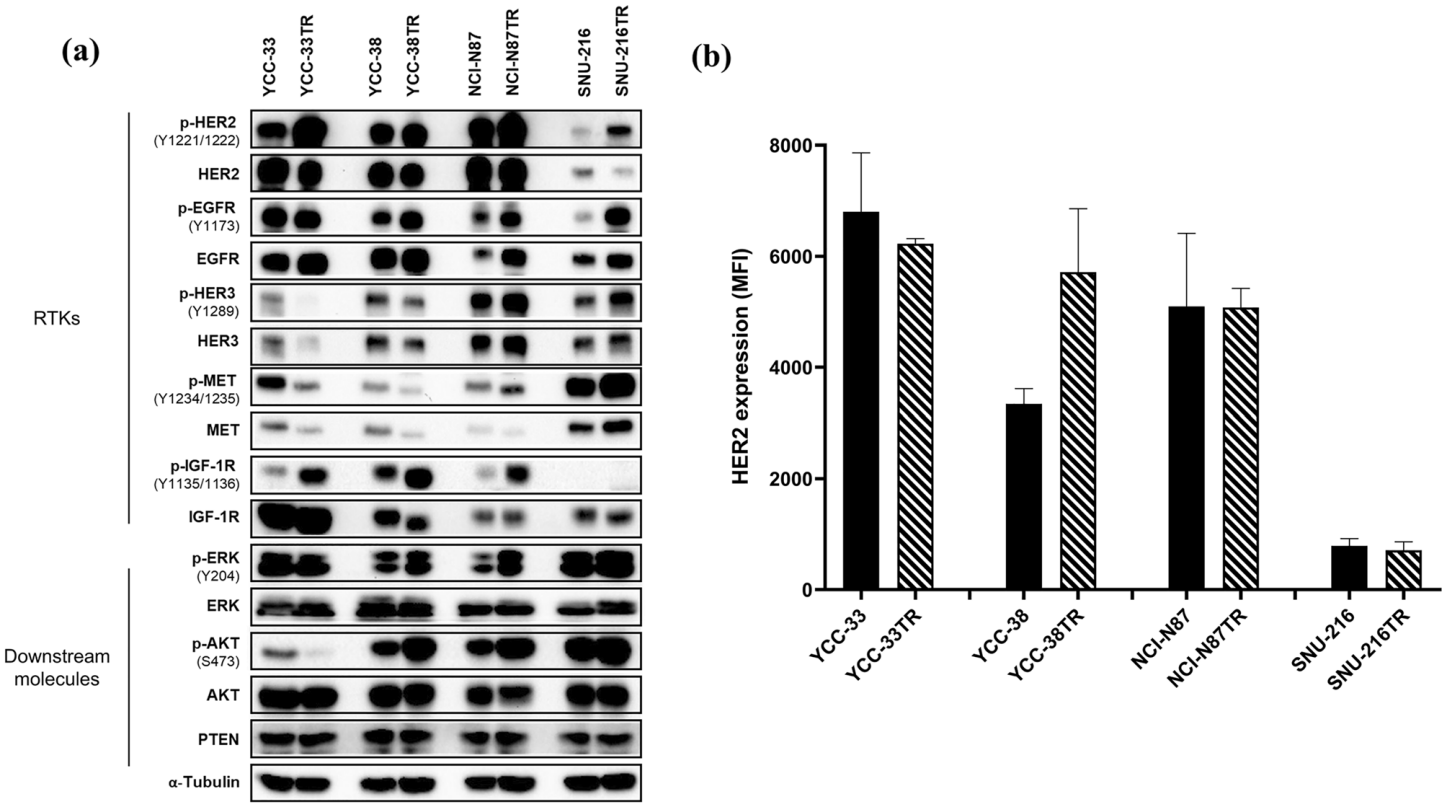
Comparative molecular profiling of the parental and trastuzumab-resistant (TR) cell lines. (**a**) Immunoblot analysis of receptor tyrosine kinases (RTKs) and related downstream molecules. α-Tubulin was used as the loading control. The blots were cut prior to hybridization with antibodies. The uncropped version of the western blots is reported in Supplementary Fig. S2. (**b**) Cell surface expression of HER2 was determined by flow cytometry (mean fluorescence intensity, MFI). Error bars, SD.

To investigate the specific molecular mechanisms responsible for trastuzumab resistance, we evaluated several RTKs and their downstream signaling pathways. Compared with the parental cell lines that had different levels of basal HER2 expression, pHER2 expression was increased in all TR cell lines, and HER2 expression varied but remained at a high level (Fig. 3a). Consistent with the level of HER2 detected in whole-cell lysates by immunoblotting, HER2 expression on the cell surface remained as high as that in the parental cell lines (Fig. 3b). Moreover, the pEGFR level was upregulated in YCC-38TR, NCI-N87TR, and SNU-216TR cells; the pHER3 level was upregulated in NCI-N87TR and SNU-216TR cells; the pMET level was upregulated in SNU-216TR cells; and the pIGF1R level was upregulated in YCC-33TR, YCC-38TR, and NCI-N87TR cells. Additionally, the pERK and pAKT levels were upregulated in YCC-38TR, NCI-N87TR, and SNU-216TR cells (Figs. 3a and Supplementary Fig. S2). Hence, the established TR cell lines maintained high HER2 expression, and each TR cell line activated various signaling pathways and developed resistance to trastuzumab through different mechanisms.

### Sensitivity to HER2-targeted agents in parental and TR cell lines

**Figure 4:**
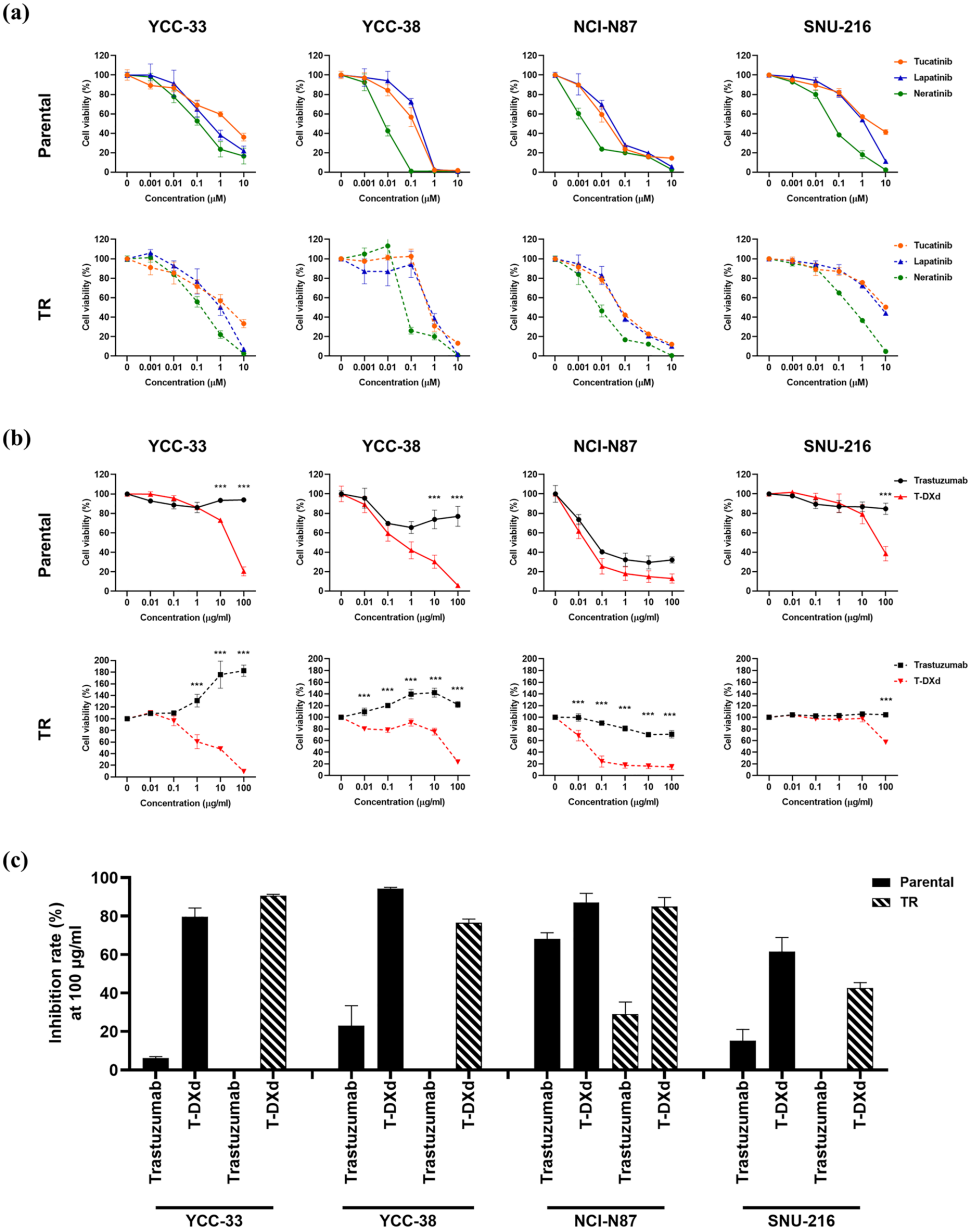
Comparative sensitivity to HER2-targeted agents in parental and trastuzumab-resistant (TR) cell lines. (**a**) Parental and TR cell lines were treated with increasing concentrations of tucatinib, lapatinib, and neratinib for 3 days. Cell viability was measured using the Cell Counting Kit-8 (CCK-8) assay. (**b**) Parental and TR cell lines were treated with increasing concentrations of trastuzumab and trastuzumab deruxtecan (T-DXd) for 6 days. Cell viability was measured using the CCK-8 assay. (**c**) Percent inhibition (inhibition rate) of trastuzumab and T-DXd at 100 μg/mL was compared between parental and TR cell lines. Error bars, SD. ***p < 0.001, **p < 0.01, *p < 0.05.

While evaluating the sensitivity of HER2-targeted agents, such as TKIs (tucatinib, lapatinib, and neratinib) and an ADC (T-DXd), both parental and TR cell lines showed sensitivity to three HER2 TKIs, with similar trends. In particular, the pan-HER inhibitor neratinib showed the highest sensitivity (Table 2, Fig. 4a and Supplementary Fig. S3). Moreover, the parental cell lines were sensitive to both trastuzumab and T-DXd. As expected, T-DXd showed superior cell growth inhibition. TR cell lines were resistant to trastuzumab but sensitive to T-DXd (Fig. 4b). The inhibition rates of T-DXd (100 μg/mL) in parental and TR cells were as follows: YCC-33 *vs*. YCC-33TR (79.6% *vs*. 90.7%), YCC-38 *vs*. YCC-38TR (94.4% *vs*. 76.5%), NCI-N87 *vs*. NCI-N87TR (87.2% *vs*. 85.0%), and SNU-216 *vs*. SNU-216TR (61.5% *vs*. 42.7%) (Fig. 4c). In addition, SN-38, an active metabolite of the topoisomerase I inhibitor irinotecan, showed similar sensitivities in all parental and TR cell lines (Supplementary Fig. S4). However, no correlation was observed between the sensitivity of T-DXd and SN-38 (data not shown), suggesting that T-DXd activity may be related to HER2 expression in addition to the sensitivity to topoisomerase I inhibition. Notably cell lines with relatively high HER2 protein expression levels, such as YCC-33, YCC-38, and NCI-N87, generally exhibited higher sensitivity to T-DXd. While SNU-216 with relatively low HER2 protein expression demonstrated lower sensitivity to T-DXd (Fig. 4c). These findings underscore the potential significance of HER2 expression levels in predicting the response to T-DXd.

**Table 2:** IC50 of T-DXd and TKIs in parental and TR cell lines.

Cell line	IC50			
	T-DXd (μg/mL)	Tucatinib (μM)	Lapatinib (μM)	Neratinib (μM)
YCC-33	17.81	2.20	0.72	0.23
YCC-33TR	9.53	1.67	0.89	0.71
YCC-38	0.53	0.06	0.08	0.01
YCC-38TR	17.00	0.37	0.20	1.44
NCI-N87	0.01	0.04	0.05	0.00
NCI-N87TR	0.01	0.11	0.12	0.01
SNU-216	10.97	3.54	0.71	0.06
SNU-216TR	12.44	10.27	7.85	0.21

Abbreviations: IC50, Half-maximal inhibitory concentration.

### Mechanism of overcoming trastuzumab resistance with T-DXd

**Figure 5:**
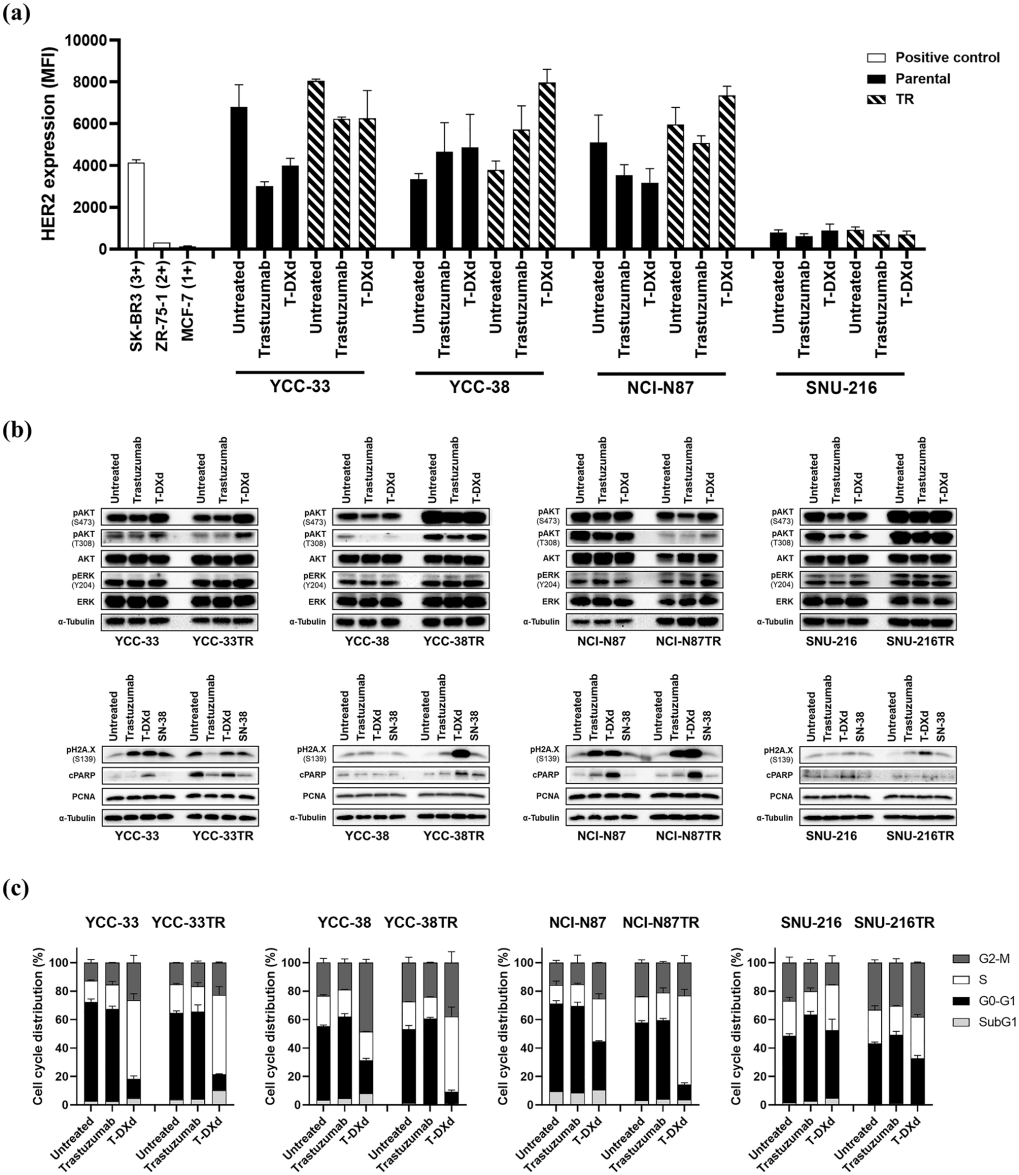
Analysis of changes in protein expression and cell cycle distribution after drug treatment in parental and trastuzumab-resistant (TR) cell lines. Trastuzumab, trastuzumab deruxtecan (T-DXd), and SN-38 were used at 200 μg/mL, 10 μg/mL, and 1 nM, respectively. (**a**) After 72 h of drug treatment, the expression level of HER2 on the cell surface was quantified by flow cytometry (mean fluorescence intensity, MFI). Three breast cancer cell lines (SK-BR-3, ZR-75–1, and MCF7) were used as controls of HER2 expression. (**b**) Top row: change in expression of pAKT-Ser473, pAKT-Thr308, AKT, pERK-Thr204, and ERK after 4 h of drug treatment; bottom row: expression of pH2A.X-Ser139, cleaved PARP, and PCNA after 72 h of drug treatment. α-Tubulin was used as the loading control. The blots were cut prior to hybridization with antibodies. The uncropped version of the western blots is reported in Supplementary Fig. S6. (**c**) Parental and TR cell lines of YCC-33, NCI-N87, and SNU-216 were treated with trastuzumab (100 μg/mL) or T-DXd (10 μg/mL) for 24 h, and YCC-38 and YCC-38TR were treated for 72 h. Cell cycle distribution was analyzed by flow cytometry. Error bars, SD.

We further investigated the potential biological mechanism of T-DXd in the parental and TR cell lines. When treated with trastuzumab or T-DXd, HER2 expression on the cell surface was decreased in YCC-33 and NCI-N87 cells and slightly increased or remained unchanged in YCC-38 and SNU-216 cells. Contrastingly, four TR cell lines showed a cell type-specific response to trastuzumab or T-DXd. In particular, the HER2 mean fluorescence intensity in YCC-38TR remarkably increased by 1.7-fold (trastuzumab) and 2.3-fold (T-DXd) (Fig. 5a and Supplementary Fig. S5).

Next, the expression of HER2 downstream signals, DNA double-strand breaks with pH2A.X, apoptosis with cPARP, and proliferation with PCNA were detected using immunoblotting. Trastuzumab or T-DXd treatment downregulated the expression of pAKT in parental cells, except for YCC-33, whereas downregulation was not observed in the TR cell lines (Fig. 5b and Supplementary Fig. S6). In YCC-33 and NCI-N87 cells, pH2A.X expression was upregulated following trastuzumab, T-DXd, and SN-38 treatments, and cPARP expression was upregulated only by T-DXd treatment. No changes were observed in YCC-38 and SNU-216. By contrast, in TR cell lines, pH2A.X expression was noticeably upregulated by T-DXd treatment, and cPARP expression was also increased in these TR cell lines, except for SNU-216TR. Interestingly, YCC-33TR, which demonstrated stimulated growth in the presence of trastuzumab, showed a decrease in the expression of pH2A.X and cPARP upon treatment with trastuzumab. PCNA expression was not affected by the drug treatments (Figs. 5b and Supplementary Fig. S6).

Flow cytometry revealed that S and G2-M arrest was significantly induced by T-DXd treatment in YCC-33 and NCI-N87 cells. In YCC-38 cells, trastuzumab and T-DXd caused G1 arrest and G2-M arrest, respectively. In SNU-216 cells, G1 arrest was induced by trastuzumab, but T-DXd showed no effect. In all TR cell lines, T-DXd significantly induced cell cycle arrest in the S or G2-M phase (Fig. 5c).

Altogether, these results demonstrate that each cell line revealed different biological responses to T-DXd treatment. When treated with trastuzumab or T-DXd, HER2 or its downstream signals were disrupted in parental cells, but not in TR cells. Interestingly, T-DXd increased the expression of pH2A.X and cPARP and caused cell cycle arrest in the S or G2-M phases in TR cell lines, suggesting increased apoptosis through DNA damage.

## Discussion

Trastuzumab can treat *HER2*-amplified metastatic GC. However, most patients who initially responded to the drug develop acquired resistance within 1 year. Molecular mechanisms driving trastuzumab resistance in GC remain largely unknown^8,9,28^. Therefore, investigating the mechanism of trastuzumab resistance may help in discovering novel molecular targets and achieving therapeutic efficacy in GC.

To date, only three GC cell lines (NCI-N87, SNU-216, and MKN-45) have been studied for trastuzumab resistance^29–31^. MKN-45 is a trastuzumab-sensitive cell line characterized by amplification of *cMET* and normal levels of HER2 expression (IHC score: 0; Table 1)^21,31,32^. However, these cell models are insufficient for understanding the underlying mechanism of trastuzumab resistance. Additionally, to evaluate acquired resistance, trastuzumab-treated cell lines are required. Thus, we established TR cell lines using six *HER2*-amplified GC cell lines, including our own patient-derived GC cell lines (YCC-19, YCC-33, and YCC-38).

Resistance to antitherapeutic drugs is primarily associated with phenotypic changes regarding cell survival, growth, migration, and invasion^33,34^. We investigated the phenotypic changes in the TR cell lines and compared them to those in parental cells. Our experiments revealed the following: (1) YCC-33TR demonstrated decreased proliferation and migration abilities, (2) YCC-38TR demonstrated increased proliferation and decreased migration and invasion abilities, (3) NCI-N87TR demonstrated increased proliferation ability, and (4) SNU-216TR demonstrated decreased proliferation ability. These results suggested that each TR cell line had different phenotypic characteristics.

The potential mechanisms underlying trastuzumab resistance in breast cancer are the activation of downstream signaling routes, such as the PI3K/AKT and MAPK/ERK pathways, and compensatory activation of signals mediated by other RTKs^3,35–37^. Trastuzumab resistance in HER2-positive breast cancer involves increased EGFR and IGF1R signals as well as dysregulation of the PTEN/PI3K/AKT/mTOR pathway^38^. MET can contribute to trastuzumab resistance in HER2-positive breast cancer cells through sustained activation of downstream signaling pathways. Loss of MET function significantly improves the response to trastuzumab, whereas MET activation protects cells against trastuzumab anticancer activity^39^. Unlike the mechanism of trastuzumab resistance in breast cancer, that of such resistance in GC remains unclear given the relative lack of research. Consistent with the results of previous studies, our data showed that various RTKs were activated, followed by the activation of downstream signaling, thus developing trastuzumab resistance in GC. Our data also provide strong evidence that each TR cell line in GC develops drug resistance through a different mechanism. Interestingly, all TR cell lines showed increased phosphorylation of HER2, and the expression of HER2 varied but remained high. HER2-targeted agents were found to be important in overcoming trastuzumab resistance.

HER2 TKIs (tucatinib, lapatinib, and neratinib) can overcome trastuzumab resistance in HER2-positive breast cancer but not in GC^40–42^. In this study, we first evaluated the sensitivity of *HER2*-amplified TR GC cell lines to these three TKIs. As expected, the parental cells were sensitive. The three TKIs exhibited good antitumor effects in TR cells, suggesting that they could overcome trastuzumab resistance in GC. Since pHER2 was expressed at high levels in the four TR cell lines, it was expected to be sensitive to TKIs targeting the HER2 active kinase domain. Furthermore, recent studies have reported that neratinib is a more potent inhibitor of proliferation across multiple cancer types than lapatinib or tucatinib^15,41^. Consistent with these findings, our data showed that neratinib was the most sensitive among the three TKIs in both *HER2*-amplified parental and TR cell lines.

We further evaluated the therapeutic potential of T-DXd in overcoming acquired trastuzumab resistance in GC. Compared to trastuzumab, T-DXd showed an improved antitumor effect in parental and TR cell lines, suggesting that it could overcome trastuzumab resistance in GC. Moreover, the efficacy of T-DXd correlated with HER2 expression levels (data not shown). The parental and TR cell lines YCC-33, YCC-38, and NCI-N87 were sensitive to T-DXd, whereas SNU-216 and SNU-216TR cell lines showed less sensitivity to T-DXd. Consistent with our findings, several studies have reported that the efficacy of T-DXd was HER2 expression-dependent both in vitro and in vivo^19,20^.

Furthermore, we investigated the mechanism of T-DXd activity in both parental and TR cell lines. In parental cells, treatment with trastuzumab and T-DXd disrupted HER2 signaling; particularly, trastuzumab-induced G1 phase arrest, whereas T-DXd induced S or G2–M phase arrest. By contrast, T-DXd did not inhibit HER2 signaling in TR cell lines but caused S or G2-M phase arrest. DXd is a camptothecin (CPT) derivative. CPT is a chemotherapeutic drug exhibiting anticancer activity by selectively inhibiting the activity of topoisomerase I, an enzyme essential for DNA replication. It is selectively cytotoxic to cells replicating DNA during the S phase, arrests cells in the G2 phase, and induces chromosomal DNA fragmentation^43^. Our results suggest that DXd, a payload of T-DXd, arrests the S or G2-M phase of the cell cycle and induces apoptosis in TR cells. Moreover, the expression of pH2A.X and cPARP in the TR cell lines was remarkably upregulated by T-DXd. To the best of our knowledge, this is the first study to identify novel differences in biological mechanisms for T-DXd efficacy in parental and TR cell lines.

Recent advances in high-throughput technologies have facilitated investigations into the relationship between genotypes and phenotypes. Further research is ongoing to explore novel mechanisms underlying trastuzumab resistance through integrative analysis of genomic, transcriptomic, and proteomic data.

In summary, we established experimental models of four TR cell lines, two of which were novel TR cell lines (YCC-33TR and YCC-38TR), to study acquired resistance to trastuzumab in GC. Each of the acquired TR cell lines exhibited different characteristics. These results may provide further novel insights into the mechanisms behind trastuzumab resistance. We also emphasize that HER2-targeted therapies are potent therapeutics to overcome resistance in GC patients, given that the established TR cell lines retain high HER2 expression.

### Supplementary Information

Supplementary Information.

## Data Availability

Data are available from the corresponding author on reasonable request.

## Abbreviations

ADC: Antibody–drug conjugate
CPT: Camptothecin
FDA: Food and drug administration
GC: Gastric cancer
IHC: Immunohistochemistry
RTK: Receptor tyrosine kinase
SICR: Song-Dang institute for cancer research
T-DXd: Trastuzumab deruxtecan
TR: Trastuzumab-resistant
TKI: Tyrosine kinase inhibitor
