# Supplementary material for: Novel HER2-targeted therapy to overcome trastuzumab resistance in HER2-amplified gastric cancer
Source: Sci Rep. 2023 Dec 19;13:22648. doi: 10.1038/s41598-023-49646-5 (PMC10730520; doi:10.1038/s41598-023-49646-5)
Supplement: Supplementary file 1 — Supplementary Information. [file 41598_2023_49646_MOESM1_ESM.docx]

**Supplementary Information**

Article title: Novel HER2-targeted therapy to overcome trastuzumab resistance in *HER2*-amplified gastric cancer

Journal name: *Scientific Reports*

Author names: Juin Park, Sun Kyoung Kang, Woo Sun Kwon, Inhye Jeong, Tae Soo Kim, Seo Young Yu, Sang Woo Cho, Hyun Cheol Chung, and Sun Young Rha

Corresponding author: Sun Young Rha

Yonsei Cancer Center

Yonsei University College of Medicine

Song-dang Institute for Cancer Research

Yonsei University Health System

Seoul, South Korea

E-mail: rha7655@yuhs.ac.

**
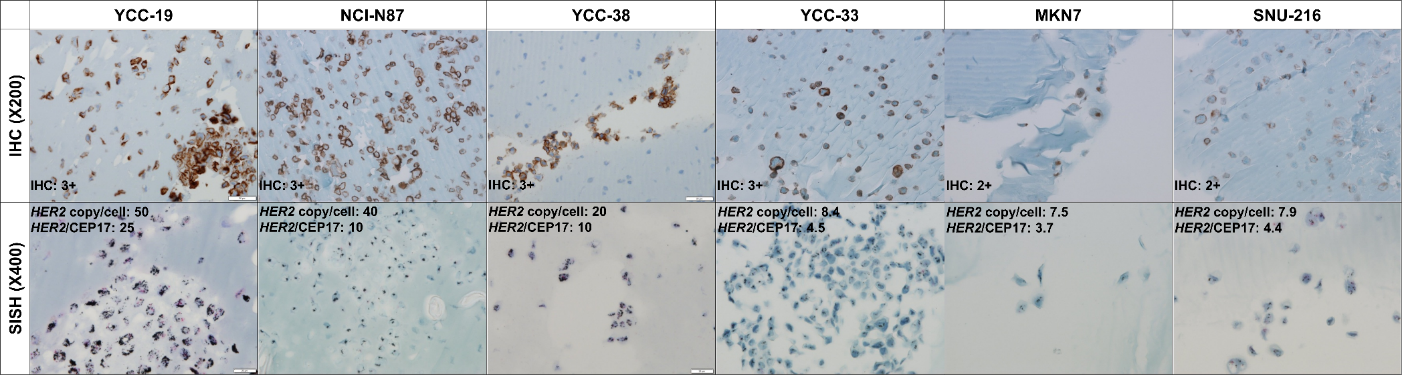
**

**Figure S1. Representative images of the HER2 status** **of six gastric cancer (GC) cell lines.** HER2 status was assessed by immunohistochemistry (IHC) and silver *in situ* hybridization (SISH).

**
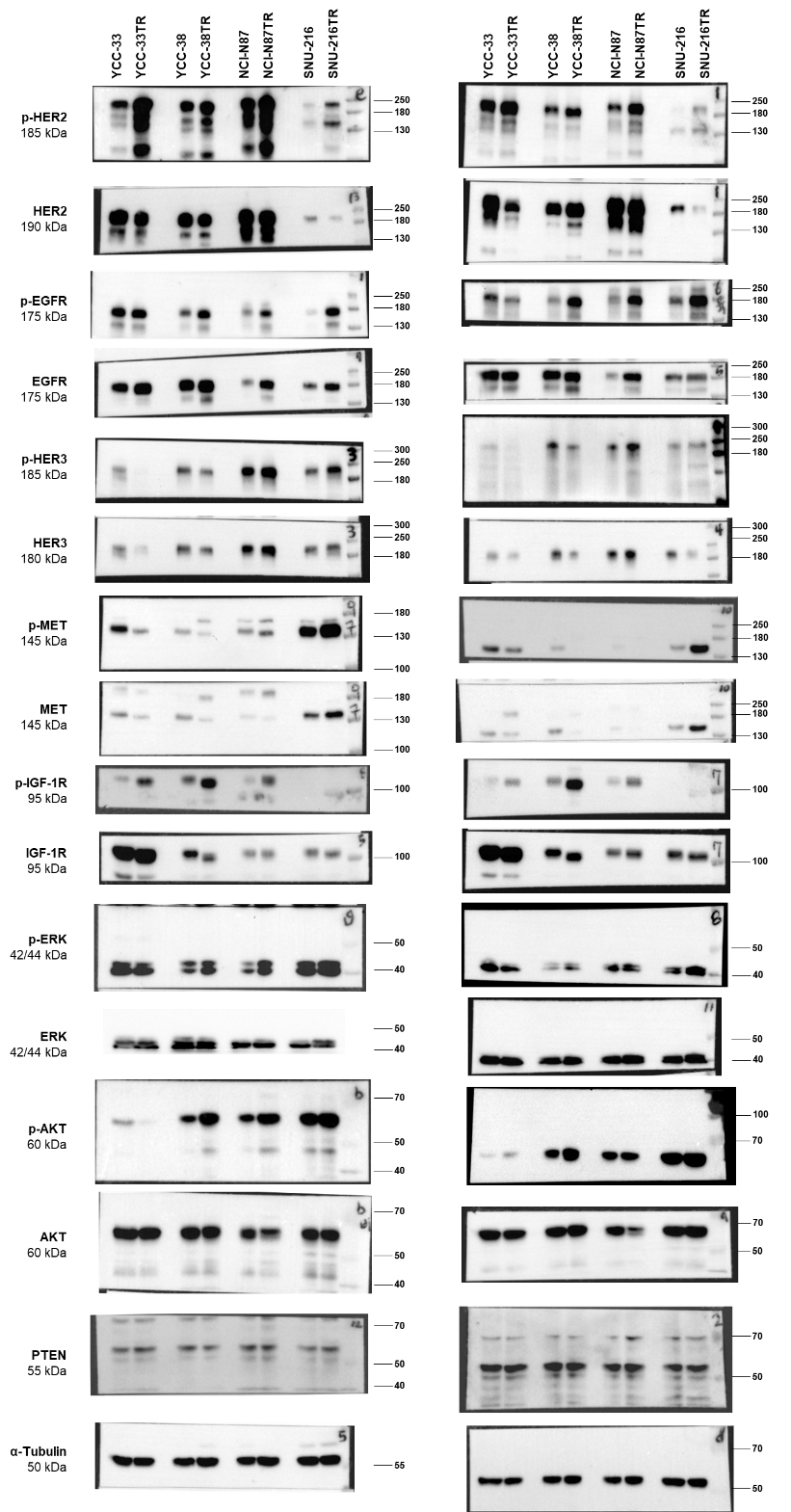
**

**Figure S2. Images of original replicated uncropped Western blots for Figure 3a.** Uncropped Western blot images are shown that correspond to Figure 3a. Molecular weight markers are indicated. Membranes were cut to enable blotting for multiple antibodies. The blots were cut prior to hybridization with antibodies and full-length blots cannot be provided.

**Figure S3. Comparative study of the inhibitory effects of tyrosine kinase inhibitors (TKIs) in parental and trastuzumab-resistant (TR) cell lines.** The inhibition rate of TKIs at 1 μM was compared between the parental and TR cell lines treated with tucatinib, lapatinib, and neratinib for 3 days. Cell viability was measured using the Cell Counting Kit-8 (CCK-8) assay. Error bars, standard deviation (SD).

**
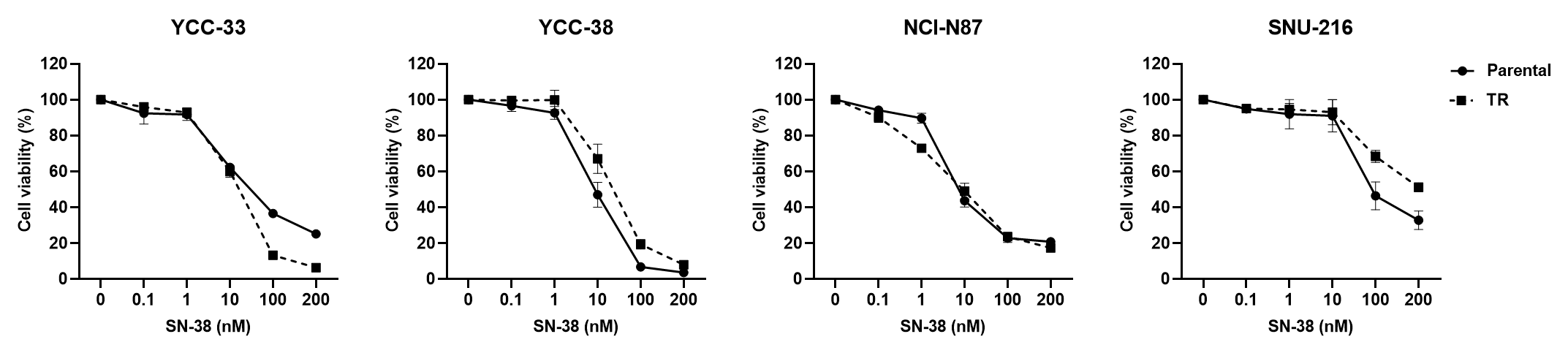
**

**Figure S4. SN-38 sensitivity of parental and TR cell lines.** Parental and TR cell lines were treated with increasing concentrations of SN-38 for 3 days. Cell viability was measured using the CCK-8 assay. Error bars, SD.


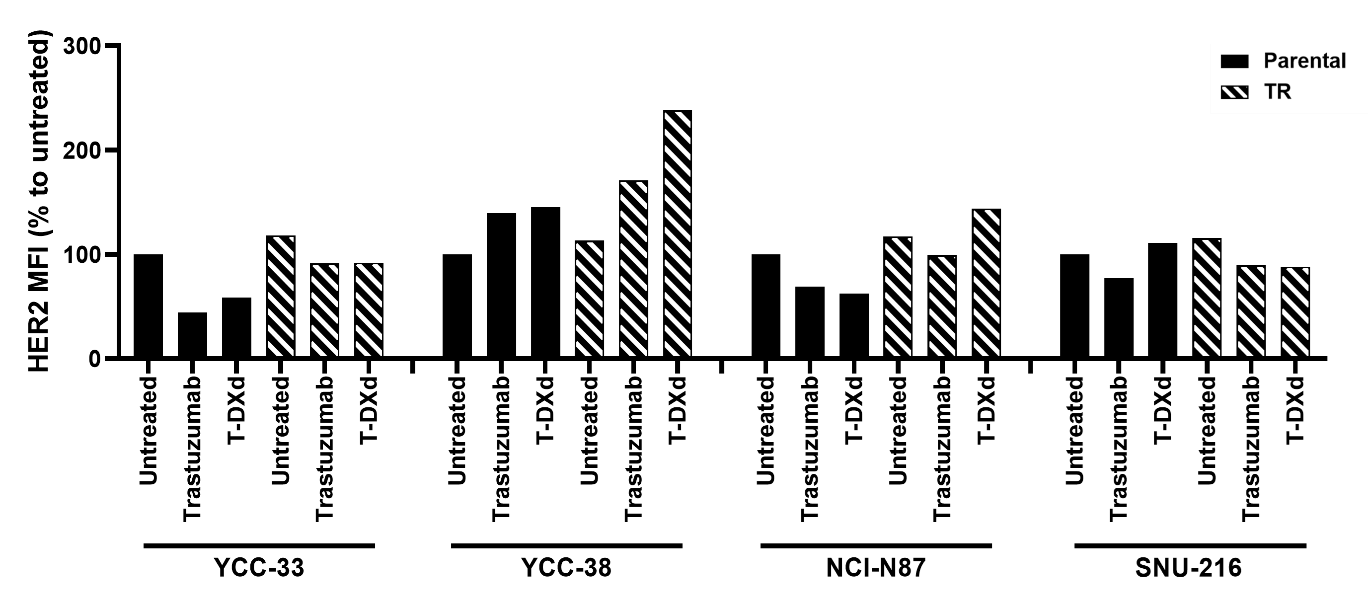


**Figure S5. Relative HER2 mean fluorescence intensity (MFI) normalized to untreated controls in each parental cell line.** Trastuzumab and trastuzumab deruxtecan (T-DXd) were used at 200 μg/mL and 10 μg/mL, respectively. After 72 h of drug treatment, the expression level of HER2 on the cell surface was quantified by flow cytometry. The relative HER2 MFI was normalized to the untreated control in each parental cell line.


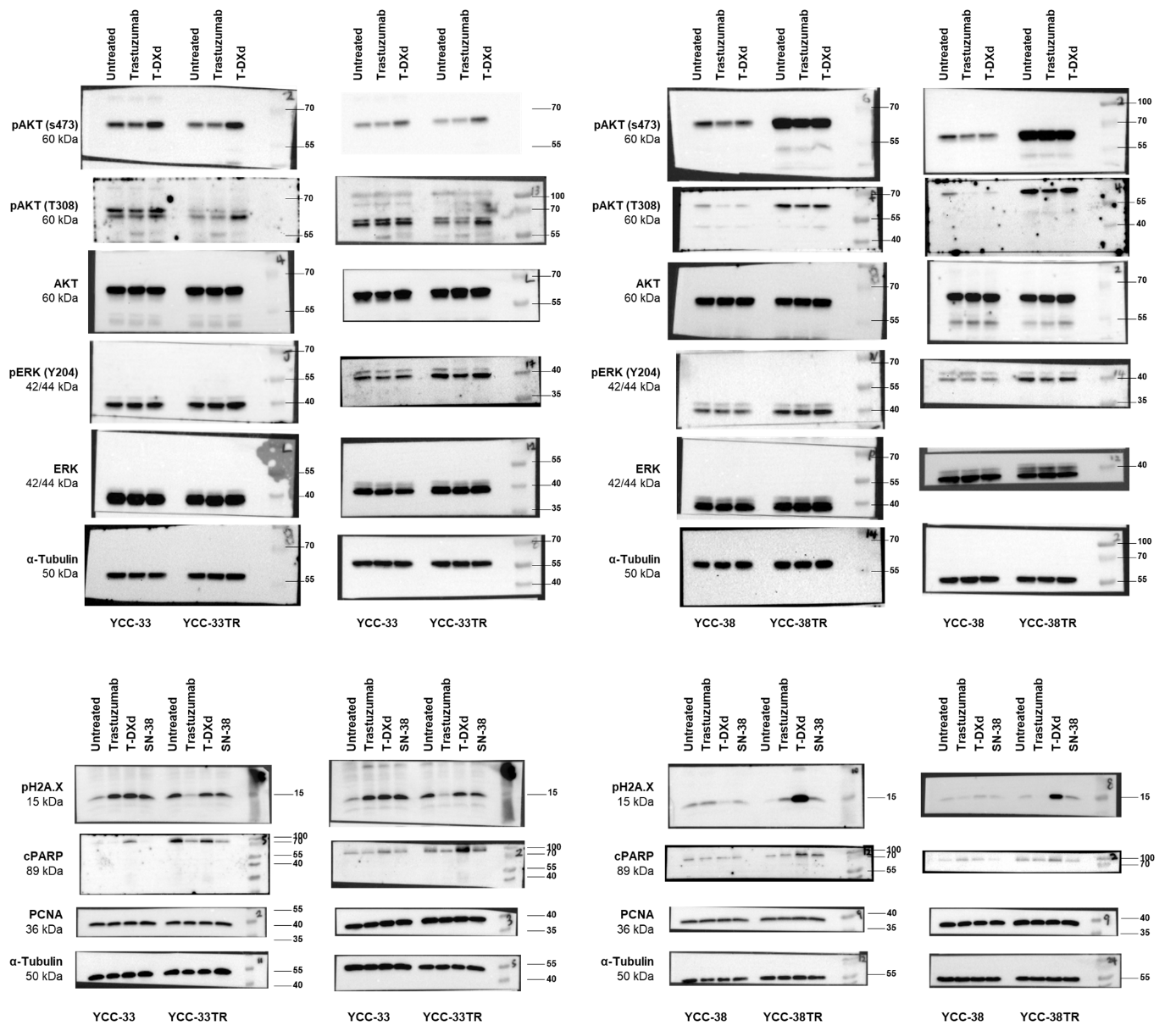


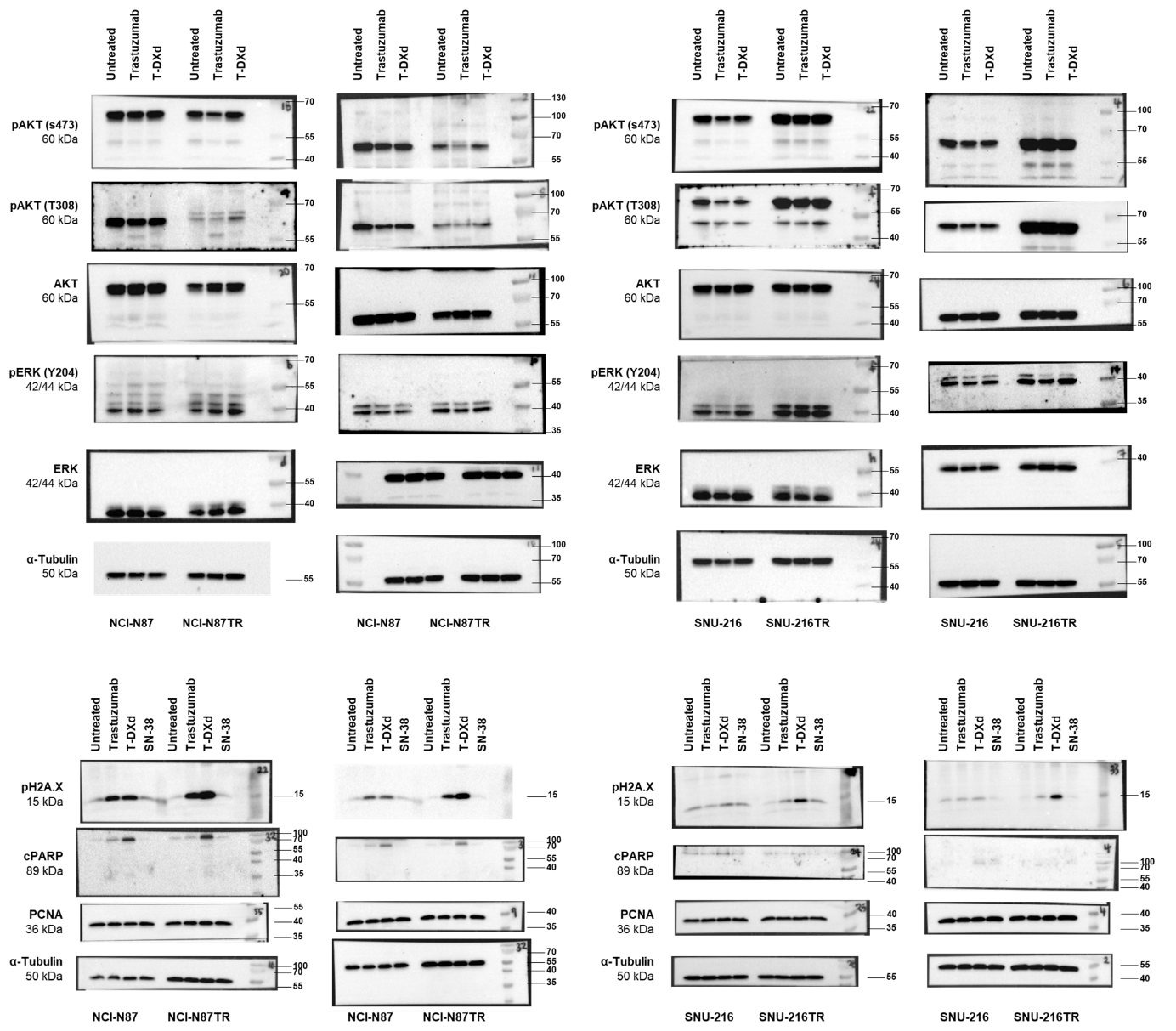


**Figure S6. Images of original replicated uncropped Western blots for Figure 5b.** Uncropped Western blot images are shown that correspond to Figure 5b. Molecular weight markers are indicated. Membranes were cut to enable blotting for multiple antibodies. The blots were cut prior to hybridization with antibodies and full-length blots cannot be provided.
